# Greater activity, better range of motion and higher quality of life following unicompartmental knee arthroplasty: a comparative case–control study

**DOI:** 10.1007/s00402-019-03296-3

**Published:** 2019-11-04

**Authors:** Georg Hauer, Patrick Sadoghi, Gerwin A. Bernhardt, Matthias Wolf, Paul Ruckenstuhl, Andrea Fink, Andreas Leithner, Gerald Gruber

**Affiliations:** Department of Orthopaedics and Trauma, Medical University of Graz, Auenbruggerplatz 5, 8036 Graz, Austria

**Keywords:** Unicompartmental knee arthroplasty, Total knee arthroplasty, Knee osteoarthritis, Quality of life

## Abstract

**Purpose:**

The purpose of this study was to provide a matched cohort comparison of clinical and functional outcome scores, range of motion and quality of life following unicompartmental knee arthroplasty (UKA) or total knee arthroplasty (TKA). The hypothesis was that patients receiving UKA report better results than comparable patients who receive conventional TKA.

**Methods:**

Clinical and functional results of 35 patients with medial end-stage osteoarthritis who had received a fixed-bearing UKA were compared with the results of 35 matched patients who had received a TKA from the same manufacturer by the same surgeon. Outcome scores were measured before surgery and at final follow-up using Tegner Activity Scale (TAS), range of motion (ROM) and Short Form 36 Health Survey (SF-36). The Knee Society Score (KSS) was assessed at final follow-up. The mean observation period was 2.3 years in both groups.

**Results:**

The preoperative knee scores had no statistically significant differences between the two groups. Postoperatively, however, UKAs performed significantly better regarding TAS and ROM (4 vs. 3 and 118.4 vs. 103.7, respectively). The results of the SF-36 showed significantly better results for the UKA group in the mental component summary score and in the subscale of social function.

**Conclusions:**

The present study suggests that UKA is associated with higher activity level, higher quality of life, and greater ROM when compared with TKA on comparable patients. Prolonged clinical follow-up in a larger patient cohort with a randomised-controlled study design would be beneficial to confirm these findings.

**Level of evidence:**

III.

## Introduction

Unicompartmental knee arthroplasty (UKA) and total knee arthroplasty (TKA) are both considered reliable and successful treatment strategies for isolated unicompartmental osteoarthritis (OA) of the knee. There is a lack of consensus, however, which of the two prostheses is to be preferred [1].

Compared to TKA, benefits of UKA include faster recovery, lower perioperative morbidity and mortality, shorter hospital stay, improved range of motion (ROM) and return to high level of sports activities [2-4]. Furthermore, both, mobile and fixed-bearing UKA closely resemble native knee kinematics thus permitting more natural joint mechanics [5, 6]. These advantages in combination with improvements in surgical techniques and implant design have resulted in rising implantation rates of UKAs over the last years [7-9].

An age under 60 years used to be a contraindication for UKA, however, the use of UKA is recently increasing and more common in patients under the age of 65 years [10, 11]. It is expected that more and more young patients come in the need for joint replacement as obesity on the one hand and sport-related injuries on the other are anticipated to increase [4]. This leads to high expectations in a physically, still active patient group [12]. Revision rates and survival, conversely, might be negatively affected by higher loads and longer patient survival [13, 14].

The choice between UKA and TKA remains challenging for surgeons and, therefore, the purpose of this case–control study was to provide comparative data about activity level, functional outcome, quality of life (QOL) and ROM in young patients following either UKA or TKA. The hypothesis was that patients receiving UKA report better results than comparable patients who receive conventional TKA.

## Patients and methods

In this retrospective comparative case–control study, the first 35 consecutive Sigma® High Performance (HP) Partial Knee System (DePuy Synthes, Warsaw, IN) UKA, performed between 2012 and 2015, were compared with a matched-control group of 35 patients following TKA. All surgeries were performed by the senior author (G.G). The Sigma® HP UKA is a fixed-bearing, cemented, medial UKA. TKA patients received the P.F.C Sigma® (DePuy Synthes, Warsaw, IN) by the same surgeon.

UKA patients were matched on a case-by-case basis to TKA patients for age, sex, BMI and follow-up period. The mean age of the 70 patients in both groups at surgery was 66 years (66 ± 8.6 UKA, 66 ± 8.1 TKA). The average length of time between surgery and last follow-up was 2.3 years in both cohorts (range 0.6–6.4 and 0.8–5.2 years, respectively). No UKA was revised or converted to TKA during the follow-up period. In the UKA group, 10 patients were male and 25 were female. 13 females and 22 received TKA. The mean BMI of the 35 patients in the UKA group at surgery was 28.7 ± 4.2 kg/m^2^. The mean BMI of the TKA patients at surgery was 28.5 ± 4.3 kg/m^2^. No significant group differences were observed concerning smoking status, alcohol consumption, educational and relationship status. Detailed results of patient demographics are given in Table 1.

**Table 1:** Patient demographics and baseline characteristics

Demographics	UKA (*n* = 35)	TKA (*n* = 35)	*p* value
Sex (M/F) (*n*/%)	10 (29%)/25(71%)	13 (37%)/23(63%)	0.611
Age (years), mean (SD)	66.0 (8.6)	66.0 (8.1)	0.973
BMI (kg/m^2^), mean (SD)	28.7 (4.2)	28.5 (4.3)	0.891
Follow-up (years), mean (SD)	2.3 (1.6)	2.3 (1.1)	0.833

Only patients with medial end-stage OA who showed no joint instability or high-grade degenerative changes of the patella surface or osteoarthritis of the contra-lateral compartment were included. Preoperatively, magnetic resonance imaging was obtained to evaluate articular cartilage of the lateral and patellofemoral joint. Exclusion criteria were flexion contracture, flexion less than 80°, varus and valgus deformities of more than 10°, lateral and patella-femoral compartment OA (Kellgren–Lawrence Grade 3–4). None of the included TKA patients was initially scheduled for UKA and intraoperatively converted to TKA. All of them refused to receive UKA prior to surgery due to personal preferences.

In all patients, a medial para-patellar approach was used and intravenous cefuroxime was administered perioperatively as single-shot antibiosis. Prior to definite decision of implantation of an UKA, the patellofemoral and lateral compartments were inspected carefully during surgery to rule out degenerative changes and cartilage defects.

In both cohorts, patients were advised to complete the same postoperative rehabilitation protocol that comprised in-patient and out-patient rehabilitation. Full weight bearing with the use of crutches was allowed to all patients immediately after surgery and continuous passive motion (CPM) therapy started on the first postoperative day. Regardless of the implant choice, only participation in low-impact sports was recommended after surgery. Sports such as running were not recommended.

Preoperatively, and at follow-up, all patients were asked to define their level of activity using the Tegner Activity Scale (TAS) [15]. The TAS assess activities of daily living, recreation and competitive sports and grades on a scale from 0 to 10. To assess knee function, the Knee Society Score (KSS) was administered [16]. The KSS includes subscales on pain and activities of daily living. ROM was assessed with a goniometer. Pre- and postoperatively, full-leg radiographic evaluation was performed to determine mechanical axes in both groups.

The Short Form 36 Health Survey (SF-36) was assessed for subjective patients’ outcome [17]. The SF-36 is a commonly used patient-reported questionnaire to survey health-related QOL. The score enables patients to quantify their health status from their own perspective and is an indicator of overall health status. The SF-36 consists of eight scaled scores including physical function (PF), role physical (RP), bodily pain (BP), vitality (VT), general health (GH), social function (SF), role emotional (RE) and mental health (MH) and two summary scores, mental component summary (MCS) and physical component summary (PCS). The score ranges from 0 to 100 points. High scores equate to good health and low scores to poor health.

This study followed accepted ethical, scientific and medical standards and was conducted in compliance with recognised international standards, including the principles of the Declaration of Helsinki. Ethical approval was obtained from the local ethical committee.

### Statistical analysis

All data were analysed by SPSS Version 22.0 (IBM Corporation, New York, USA). Descriptive statistics for continuous variables were reported as the mean and standard deviation (SD) and the median and range. Categorical variables were reported as count and proportions. For comparisons of categorical variables, the Chi square exact test was used. Data were tested for normality using Kolmogorov–Smirnov test, which revealed a non-parametric distribution. Differences between preoperative and postoperative data were observed with Mann–Whitney *U* test and Wilcoxon signed-rank test. A *p* value of < 0.05 was defined as statistically significant. Post hoc power analysis was calculated according to Hoenig and Heisey with respect to the magnitude of differences in all clinical scores and ROM [18].

## Results

### Pre- and postoperative assessment of activity level, function and range of motion

The TAS was obtained immediately preoperatively and after a mean follow-up of 2.3 years. Preoperatively, the median TAS did not differ among the study groups, whereas at last follow-up, UKA patients showed a significantly higher (*p* = 0.021) median activity level. The change in score (preoperatively to latest postoperatively) demonstrated a significant (*p* < 0.001) difference in favour of UKA patients.

The mean KS clinical score was higher (*p* < 0.001) for the UKA group than for the TKA group (96.9 ± 5.2 vs. 91.3 ± 10.1), whereas the functional score was similar in both groups (93.1 ± 12.1 vs. 89.8 ± 12.6). At final follow-up, the mean ROM for the UKA group was significantly better (*p* < 0.001) than for the TKA group (118.4 ± 10.0 vs. 103.7 ± 19.6), whereas mean values did not differ before surgery (105.4 ± 17.6 vs. 107.7 ± 12.3).

All details are given in Table 2.

**Table 2:** Comparison of clinical outcome before (except KSS) and after mean final follow-up (2.3 years)

	UKA (*n* = 35)	TKA (*n* = 35)	
KSS pain (mean ± SD)	96.9 ± 5.2	91.3 ± 10.1	***p* < 0.001**
KSS function (mean ± SD)	93.1 ± 12.1	89.8 ± 12.6	*p* = 0.107
ROM (°) (mean ± SD)			
Preoperative	105.4 ± 17.6	107.7 ± 12.3	*p* = 0.531
Postoperative	118.4 ± 10.0	103.7 ± 19.6	***p* < 0.001**
Change in ROM	13.0 ± 19.8	4.0 ± 22.8	***p* = 0.001**
TAS [median(min–max)]			
Preoperative	2 (1–3)	2 (0–5)	*p* = 0.163
Postoperative	4 (2–6)	3 (2–6)	***p* = 0.021**
Change in score	2 (1–3)	1 (0–2)	***p* < 0.001**
Mechanical axis (°) (mean ± SD)			
Preoperative	Varus 5.9 ± 4.2	Varus 6.2 ± 4.2	*p* = 0.795
Postoperative	Varus 3.6 ± 2.6	Varus 1.7 ± 2.3	***p* = 0.016**

All statistically significant differences are highlighted in bold font

*SD* standard deviation, *UKA* unicompartmental knee arthroplasty, *TKA* total knee arthroplasty, *KSS* Knee Society Score, *ROM* range of motion, *TAS* Tegner Activity Scale

### SF-36 health survey

The results of the SF-36 showed significantly (*p* = 0.037) better results for the UKA group in the mental component summary score (49.4 ± 9.5 vs. 45.3 ± 6.7). Furthermore, significantly (*p* = 0.017) better results were found in the subscale of social function (90.7 ± 14.0 vs. 80.4 ± 20.9). No significant differences were measured in the subscales as well as in the summary of physical components between the two study groups.

The selected sample size of *n* = 35 per group was sufficient in the analysis. Observed power was calculated greater than 80% according to Hoenig and Heisey with respect to the difference in the included clinical scores and ROM, revealing a *p* value < 0.01 [18]

## Discussion

The aim of the study was to compare the outcome regarding functional abilities, activity level, ROM and QOL following Sigma HP UKA and P.F.C Sigma TKA. To our knowledge, this is the first case–control study to compare the functional outcome and QOL between those two procedures. The most important finding of the study is that patients receiving Sigma HP UKA have reported better results than comparable patients who received conventional P.F.C Sigma TKA. Significantly better ROM, better clinical performance and higher activity levels and QOL were observed during the follow-up period for the UKA. No statistically significant difference was observed for the functional component of the KSS for either group.

Since most of the favourable clinical outcome following UKA results from medial mobile-bearing designs, it was necessary to provide additional data concerning a medial fixed-bearing UKA. Several studies have already been carried out to provide data following medial fixed-bearing UKA with similar findings. Argenson et al. reported almost identical KS clinical and functional scores at a mean follow-up of 20 years with an average of 91 and 88 points, respectively [19]. Additionally, Winnock de Grave et al. in a retrospective study with a mean follow-up of 5.5 years found excellent or good outcomes in almost 95% of their patients after medial fixed-bearing UKA [20]. A more recent study has analysed the role of a fixed-bearing UKA in patients with severe angular deformities: even then, similar ROM and improvements in SF-36 could be achieved after 2 years [21].

Patients’ expectations concerning activity level after knee arthroplasty can be high and partly unrealistic. Surgeons should consider various variables before recommendations for either TKA or UKA can be given. Preoperative scoring systems, like the Unicompartmental indication score (UIS), can be used to aid proper patient selection [22]. Moreover, preoperative lifestyle, sport level and patient’s motivation should be known to fulfil patients’ expectation. Low-impact sports such as hiking, swimming and cycling are generally recommended after knee arthroplasty [3, 14, 23]. However, high impact sports with running and jumping sequences can increase the risk of wear, implant loosening and periprosthetic fractures and can only be advised to properly selected patients [13, 14]. Several studies have shown higher sports levels and quicker return-to-sport rates for patients undergoing UKA compared to TKA [4, 12, 14, 24, 25]. This present study found significantly superior results for postoperative Tegner score in patients receiving UKA, although preoperative values were equally distributed among the study groups. As every single step from 1 to 10 consists of a large bandwidth of activities, increases from one point to another relatively implies great improvements [12].

Functional outcome measurement is an important tool when comparing UKA and TKA as comparison of revision rates might lead to misinterpretation of performance. Several systematic reviews and national arthroplasty registers have shown higher revision rates for UKA than for TKA [4, 7-9, 26]. Nevertheless, few things have to be considered using risk of reoperation as the outcome. UKAs are generally performed in a younger, more active population group and return-to-sport rates are higher compared to TKA. However, high levels of activity increase the risk of implant loosening due to production of wear and might affect survival rates adversely [14]. Additionally, UKAs might be more likely revised and converted to a TKA in the presence of unexplained pain, as this reflects the logically next therapeutic step from a partial to a definitive solution [27, 28]

There was no difference in the postoperative functional KSS between the two groups. These findings are consistent with results from a systematic review by Kleeblad et al. [4]. The authors found that OKS, HSS and WOMAC scores were higher following UKA than TKA, whereas KSS scores did not differ among the groups [4]. One possible explanation for this was provided by Na et al. [29], who criticised the modality of the KSS, which assess only walking and stair climbing and fails to address more demanding physical activities. Accordingly, the KSS fails to differentiate the functional status in high active young patients, a main target group of UKA [29]. Still, the results of the KSS functional score in the present study were better in the UKA group, which might be clinically relevant.

The functional component of different scoring systems was already found to be adversely influenced by increasing age and elevated pain scores [30, 31]. By matching the study groups with adjustment for age, the risk of biased results in favour of a younger cohort was reduced. Concerning the clinical component of the KSS, which includes assessment of pain, a significantly postoperative difference was observed among the groups. Higher postoperative pain scores in the TKA group, consequently, might have biased functional performance. Jacobs et al. found that function scores are more related to pain scores than to objective functional performance tests after surgery. The authors suggested conducting objective measures of function to fully understand a patient’s recovery [31].

Improvement in function is well documented after both procedures but without any definite greater benefit for one or the other intervention [32]. In their database analysis, Lyons et al. revealed higher pre- and postoperatively absolute functional outcome scores for patients undergoing UKA. The change in scores, however, was similar in both groups over time [32]. Lim and colleagues also found no statistically significant difference in the change of function scores over time for both UKA and TKA [33]. Consistent with that, the other authors confirmed an association between better preoperative scores and better postoperative scores [34, 35]. By examining patient-reported outcome measures from the National Joint Registry of England and Wales (NJR), no difference was found in the improvement of either knee-specific or general health outcomes between UKA and TKA in a large cohort [36]. Goh and colleagues found no difference in functional outcome, QOL and satisfaction between UKA and TKA in patients younger than 55 years. According to them, this oft-cited benefit of UKA by preserving native biomechanics did not translate into a higher rate of satisfaction [2]. Patient-reported data regarding pain and function collected from a Norwegian Arthroplasty Register study showed only small or no differences of patients who underwent UKA and TKA [37].

An accepted and confirmed goal of UKAs is to achieve greater ROM than in TKAs [2, 4, 37, 38]. The mean postoperative flexion of UKA compared to TKA in this study was significantly higher in the UKA group at 2.3-year follow-up. The present findings are supported by a recent systematic review that showed significantly greater ROM in patients undergoing UKA at mid- to long-term follow-up [4]. Goh et al. reported equal results with significantly greater flexion at 6 months and 2 years after UKA [2]. When comparing UKA and TKA, a better ability to bend the knee after UKA with level of clinical significance was published in a Norwegian arthroplasty registry study [37]. The authors suggested a preference for UKA in patients with special need for greater range of motion [37]. Lombardi et al. discovered a significantly greater mean ROM for the UKA group after a mean follow-up of 31 months whereas the improvement from preoperative level was not different among the groups [38].

UKA and TKA are both effective treatment options in improvement of patients’ mental health status after surgery [2, 39]. One possible explanation might be that postoperative pain release prevents the onset of depressive behaviour [40]. It was also found that preoperative mental health status plays a role in patients’ satisfaction postoperatively. Lower scores were associated with patient dissatisfaction and unfulfilled expectations [41, 42]. Within this study, significantly better results were found for the UKA group in the mental component summary score. This might contribute to higher willingness and motivation during rehab, which can in turn cause superior clinical and functional scores as observed in this study [43].

One drawback of the study is the relatively small sample size and the retrospective design. More strength of results is obtained with a randomised, prospective trial. Further limitations of the study were that preoperative data about KSS were not available. Therefore, a comparison between pre- and postoperative data was not possible. The change in score, however, indicates the effect of the intervention [32].

Another limitation is that only a short to mid-term follow-up is available now and patients need to be followed further on to prove whether the superior findings for UKA remain consistent in the long term. Arthritic changes in the remainder of the joint might lead to impaired results over time in UKA.

## Conclusion

The present study suggests that Sigma HP UKA is associated with higher activity level, higher QOL, and greater ROM when compared with P.F.C Sigma TKA. Postoperatively, comparable improvement regarding KSS was achieved in both intervention groups. Prolonged clinical follow-up in a larger patient cohort with a randomised controlled study design would be beneficial to confirm these findings.

## References

[1] Koppens D, Stilling M, Munk S, Dalsgaard J, Rytter S, Sorensen OG (2018). Low implant migration of the SIGMA((R)) medial unicompartmental knee arthroplasty. Knee Surg Sports Traumatol Arthrosc.

[2] Goh GS, Bin Abd Razak HR, Tay DK, Chia SL, Lo NN, Yeo SJ (2018). Unicompartmental knee arthroplasty achieves greater flexion with no difference in functional outcome, quality of life, and satisfaction vs total knee arthroplasty in patients younger than 55 years. A propensity score-matched cohort analysis. J Arthroplasty.

[3] Jahnke A, Mende JK, Maier GS, Ahmed GA, Ishaque BA, Schmitt H (2015). Sports activities before and after medial unicompartmental knee arthroplasty using the new Heidelberg sports activity score. Int Orthop.

[4] Kleeblad LJ, van der List JP, Zuiderbaan HA, Pearle AD (2018). Larger range of motion and increased return to activity, but higher revision rates following unicompartmental versus total knee arthroplasty in patients under 65: a systematic review. Knee Surg Sports Traumatol Arthrosc.

[5] Peersman G, Slane J, Vuylsteke P, Fuchs-Winkelmann S, Dworschak P, Heyse T (2017). Kinematics of mobile-bearing unicompartmental knee arthroplasty compared to native: results from an in vitro study. Arch Orthop Trauma Surg.

[6] Catani F, Benedetti MG, Bianchi L, Marchionni V, Giannini S, Leardini A (2012). Muscle activity around the knee and gait performance in unicompartmental knee arthroplasty patients: a comparative study on fixed- and mobile-bearing designs. Knee Surg Sports Traumatol Arthrosc.

[7] No authors listed (2017) Annual report 2017. Swedish Knee Arthroplasty Register https://www.myknee.se/pdf/SVK_2017_Eng_1.0.pdf. Accessed 2 Sept 2019

[8] No_authors_listed (2017) Hip, Knee & Shoulder Arthroplasty annual report 2017 https://aoanjrr.sahmri.com/documents/10180/275066/Hip%2C%20Knee%20%26%20Shoulder%20Arthroplasty. Accessed 2 Sept 2019

[9] No authors listed (2017) National joint registry for England, Wales, Northern Ireland and the Isle of Man 14th Annual Report. https://www.njrreports.org.uk/Portals/0/PDFdownload/NJR%2014th%20Annual%20Report%202017.pdf. Accessed 2 Sept 2019

[10] Kozinn SC, Scott R (1989). Unicondylar knee arthroplasty. J Bone Joint Surg Am.

[11] A W-Dahl O Robertsson L Lidgren L Miller D Davidson S Graves 2010 Unicompartmental knee arthroplasty in patients aged less than 65 Acta Orthop 81 90 94 10.3109/17453671003587150 PMC2856210 20175656

[12] Waldstein W, Kolbitsch P, Koller U, Boettner F, Windhager R (2017). Sport and physical activity following unicompartmental knee arthroplasty: a systematic review. Knee Surg Sports Traumatol Arthrosc.

[13] Witjes S, Gouttebarge V, Kuijer PP, van Geenen RC, Poolman RW, Kerkhoffs GM (2016). Return to sports and physical activity after total and unicondylar knee arthroplasty: a systematic review and meta-analysis. Sports Med.

[14] Naal FD, Fischer M, Preuss A, Goldhahn J, von Knoch F, Preiss S (2007). Return to sports and recreational activity after unicompartmental knee arthroplasty. Am J Sports Med.

[15] Tegner Y, Lysholm J (1985). Rating systems in the evaluation of knee ligament injuries. Clin Orthop Relat Res.

[16] Insall JN, Dorr LD, Scott RD, Scott WN (1989). Rationale of the knee society clinical rating system. Clin Orthop Relat Res.

[17] Ware JE, Sherbourne CD (1992). The MOS 36-item short-form health survey (SF-36). I. Conceptual framework and item selection. Med Care.

[18] Hoenig JM, Heisey DM (2001). The abuse of power: the pervasive fallacy of power calculations for data analysis. Am Statistican.

[19] Argenson JN, Blanc G, Aubaniac JM, Parratte S (2013). Modern unicompartmental knee arthroplasty with cement: a concise follow-up, at a mean of twenty years, of a previous report. J Bone Joint Surg Am.

[20] Winnock de Grave P, Barbier J, Luyckx T, Ryckaert A, Gunst P, Van den Daelen L (2018). Outcomes of a fixed-bearing, medial, cemented unicondylar knee arthroplasty design: survival analysis and functional score of 460 cases. J Arthroplasty.

[21] Seng CS, Ho DC, Chong HC, Chia SL, Chin PL, Lo NN (2017). Outcomes and survivorship of unicondylar knee arthroplasty in patients with severe deformity. Knee Surg Sports Traumatol Arthrosc.

[22] Antoniadis A, Dimitriou D, Canciani JP, Helmy N (2019). A novel preoperative scoring system for the indication of unicompartmental knee arthroplasty, as predictor of clinical outcome and satisfaction. Arch Orthop Trauma Surg.

[23] Pietschmann MF, Wohlleb L, Weber P, Schmidutz F, Ficklscherer A, Gulecyuz MF (2013). Sports activities after medial unicompartmental knee arthroplasty Oxford III-what can we expect?. Int Orthop.

[24] Walker T, Gotterbarm T, Bruckner T, Merle C, Streit MR (2015). Return to sports, recreational activity and patient-reported outcomes after lateral unicompartmental knee arthroplasty. Knee Surg Sports Traumatol Arthrosc.

[25] Hopper GP, Leach WJ (2008). Participation in sporting activities following knee replacement: total versus unicompartmental. Knee Surg Sports Traumatol Arthrosc.

[26] Liddle AD, Judge A, Pandit H, Murray DW (2014). Adverse outcomes after total and unicompartmental knee replacement in 101,330 matched patients: a study of data from the national joint registry for England and Wales. Lancet.

[27] Scott CEH, Wade FA, MacDonald D, Nutton RW (2018). Ten-year survival and patient-reported outcomes of a medial unicompartmental knee arthroplasty incorporating an all-polyethylene tibial component. Arch Orthop Trauma Surg.

[28] Goodfellow JW, O'Connor JJ, Murray DW (2010). A critique of revision rate as an outcome measure: re-interpretation of knee joint registry data. J Bone Joint Surg Br.

[29] Na SE, Ha CW, Lee CH (2012). A new high-flexion knee scoring system to eliminate the ceiling effect. Clin Orthop Relat Res.

[30] Bremner-Smith AT, Ewings P, Weale AE (2004). Knee scores in a 'normal' elderly population. Knee.

[31] Jacobs CA, Christensen CP (2009). Correlations between knee society function scores and functional force measures. Clin Orthop Relat Res.

[32] Lyons MC, MacDonald SJ, Somerville LE, Naudie DD, McCalden RW (2012). Unicompartmental versus total knee arthroplasty database analysis: is there a winner?. Clin Orthop Relat Res.

[33] Lim JW, Cousins GR, Clift BA, Ridley D, Johnston LR (2014). Oxford unicompartmental knee arthroplasty versus age and gender matched total knee arthroplasty—functional outcome and survivorship analysis. J Arthroplasty.

[34] Vielgut I, Leitner L, Kastner N, Radl R, Leithner A, Sadoghi P (2016). Sports activity after low-contact-stress total knee arthroplasty—a long term follow-up study. Sci Rep.

[35] Burn E, Sanchez-Santos MT, Pandit HG, Hamilton TW, Liddle AD, Murray DW (2018). Ten-year patient-reported outcomes following total and minimally invasive unicompartmental knee arthroplasty: a propensity score-matched cohort analysis. Knee Surg Sports Traumatol Arthrosc.

[36] Baker PN, Petheram T, Jameson SS, Avery PJ, Reed MR, Gregg PJ (2012). Comparison of patient-reported outcome measures following total and unicondylar knee replacement. J Bone Joint Surg Br.

[37] Lygre SH, Espehaug B, Havelin LI, Furnes O, Vollset SE (2010). Pain and function in patients after primary unicompartmental and total knee arthroplasty. J Bone Joint Surg Am.

[38] Lombardi AV, Berend KR, Walter CA, Aziz-Jacobo J, Cheney NA (2009). Is recovery faster for mobile-bearing unicompartmental than total knee arthroplasty?. Clin Orthop Relat Res.

[39] ND Clement D MacDonald R Burnett (2013) Primary total knee replacement in patients with mental disability improves their mental health and knee function: a prospective study Bone Joint J 95-B 360–366 10.1302/0301-620X.95B3.29563 23450021

[40] Perez-Prieto D, Gil-Gonzalez S, Pelfort X, Leal-Blanquet J, Puig-Verdie L, Hinarejos P (2014). Influence of depression on total knee arthroplasty outcomes. J Arthroplasty.

[41] Bourne RB, Chesworth BM, Davis AM, Mahomed NN, Charron KD (2010). Patient satisfaction after total knee arthroplasty: who is satisfied and who is not?. Clin Orthop Relat Res.

[42] Lee M, Huang Y, Chong HC, Ning Y, Lo NN, Yeo SJ (2016). Predicting satisfaction for unicompartmental knee arthroplasty patients in an asian population. J Arthroplasty.

[43] Goh GS, Liow MHL, Pang HN, Tay DK, Lo NN, Yeo SJ (2018). Patients with poor baseline mental health undergoing unicompartmental knee arthroplasty have poorer outcomes. J Arthroplasty.

